# Nuestra Experiencia en Pacientes Pediátricos a los que se les ha Retirado Tratamiento Farmacológico de la Epilepsia y Siguen sin Tratamiento

**DOI:** 10.31083/RN37196

**Published:** 2025-05-27

**Authors:** Javier López Pisón, Candela Gómez Sánchez, Patricia Morte Coscolín, Maria Violeta Fariña Jara, Fernando Martínez Calvo, Ruth Fernando Martínez

**Affiliations:** ^1^Sección de Neuropediatría, Hospital Universitario Miguel Servet, 50009 Zaragoza, España; ^2^Servicio de Pediatría, Hospital Universitario Miguel Servet, 50009 Zaragoza, España; ^3^Sección de Neuropediatría, Hospital Parc Taulí, 08208 Sabadell, Barcelona, España; ^4^Sección de Neuropediatría, Hospital Quirón Salud, 50009 Zaragoza, España

**Keywords:** epilepsia, fármacos anticrisis epilépticas, trastornos del neurodesarrollo, pediatría, retirada de fármacos anticrisis epilépticas, epilepsy, anti-seizure drugs, development disorders, paediatrics, withdrawal of anti-seizure drugs

## Abstract

**Introducción:**

Existen muchas variables a considerar en la retirada del tratamiento anticrisis epilépticas, que precisa una evaluación riesgo-beneficio.

**Sujetos y Métodos:**

Estudio retrospectivo en pacientes de una consulta de neuropediatría a los que se les suprimió el tratamiento farmacológico de la epilepsia y continúan sin tratamiento.

**Resultados:**

De 57 niños a quienes se retiró tratamiento, 34 siguen sin tratamiento. En 23 se retiró una vez, con tiempo medio sin crisis hasta la retirada de 21 meses y una edad media de 10,5 años. Tres casos presentaron crisis, pero no se reintrodujo tratamiento; el tiempo medio sin crisis había sido de 44,78 meses. Se retiró el tratamiento 2 veces en 9 niños y 3 veces en 2, con tiempo medio sin crisis hasta la retirada de 28,5 meses; la edad media fue de 16,3 años. Dos casos presentaron crisis, pero no se reintrodujo tratamiento; tiempo medio sin crisis fue de 5,7 años. Se dejaron sin fármaco anticrisis epilépticas casos con riesgo alto de seguir presentando crisis: 7 casos de discapacidad intelectual, 1 epilepsia refractaria, 2 epilepsia de inicio en la adolescencia y en 11 niños y 13 ocasiones de fallo en retirada previa.

**Discusión:**

La indicación y mantenimiento del tratamiento con fármacos anticrisis en niños deben priorizar el bien del paciente y deben estar basados en tres premisas: que esté indicado, que sea tolerado y que sea eficaz. La decisión de retirada debe ser individualizada, conociendo el riesgo de recaída, atendiendo a eficacia y tolerancia, especialmente en niños con trastornos del comportamiento y neurodesarrollo.

## Abstract (en)

**Introduction:**

There are many variables to consider when withdrawing anti-epileptic seizure treatment and risk-benefit evaluation is required.

**Materials and Methods:**

Retrospective study of patients from a neuropediatric clinic who were discontinued from pharmacological treatment for epilepsy and continued without treatment.

**Results:**

Of 57 children from whom treatment was withdrawn, 34 remained without treatment. In 23 cases, treatment was withdrawn once, with a mean seizure-free time until withdrawal of 21 months and a mean age of 10.5 years. Three cases presented seizures but treatment was not reintroduced; the average time without a crisis was 44.78 months. Treatment was withdrawn twice in nine children and three times in two children, with a mean seizure-free time until withdrawal of 28.5 months; the average age was 16.3 years. Two cases presented seizures but treatment was not reintroduced; the average time without a crisis was 5.7 years. High-risk cases of recurrent epileptic seizures were left without anti-seizure medication: seven cases of intellectual disability, one refractory epilepsy, two cases of epilepsy with onset in adolescence, and in 11 children, 13 instances of failure in previous withdrawal attempts.

**Discussion:**

The indication and maintenance of treatment with anti-seizure drugs in children must prioritize patient welfare and be based on three premises: the treatment is indicated, the treatment is tolerated, and the treatment is effective. The decision to withdraw must be made on a case-by-case basis, acknowledging the risk of relapse and taking into account efficacy and tolerance, especially in children with behavioral and neurodevelopmental disorders.

## 1. Introducción

Casi el 70% de los pacientes con epilepsia entran en una remisión de las crisis durante el tratamiento con fármacos anticrisis epilépticas (FACE) [1], lo que puede plantear su retirada.

La retirada del FACE precisa de una evaluación riesgo-beneficio: recurrencia de las convulsiones y sus consecuencias frente a los efectos secundarios de los FACE (calidad percibida de vida, siendo especial en los niños sobre el neurodesarrollo, comportamiento y funciones cognitivas, así como la potencial teratogenicidad en mujeres en edad fértil).

En un trabajo previó se analizó nuestra experiencia en una consulta de neuropediatría sobre niños a los que, tras haberse retirado el FACE, se había reintroducido y permanecían en tratamiento [2].

En este trabajo se analiza nuestra experiencia en una consulta de neuropediatría en niños a los que se ha retirado el FACE y no se ha vuelto a introducir.

## 2. Sujetos y Métodos

Se trata de un estudio retrospectivo con finalidad descriptiva realizado con los datos de una consulta de neuropediatría de un hospital de referencia regional. En octubre 2017 los niños fueron distribuidos entre los distintos neuropediatras de la unidad. Criterios de inclusión: pacientes de la consulta del primer firmante a los que se les retiró el tratamiento farmacológico de las crisis antes de septiembre 2023 y seguían sin FACE en el momento de los últimos datos conocidos. Criterios de exclusión: que hubieran vuelto a tomar FACE. Este proyecto cuenta con la aprobación del Comité de Ética de Investigación local.

## 3. Resultados

Hasta septiembre de 2023 habían sido introducidos datos de 2865 pacientes, de los cuales 282 estaban diagnosticados de epilepsia. A 57 se les había retirado el FACE en algún momento, de los cuales, en el momento de los últimos datos conocidos 34 seguían sin tratamiento (2 tras 3 y 9 tras 2 intentos de retirada y 23 tras 1 sola retirada) y 23 estaban tomando FACE (17 tras 1 intento de retirada, 5 tras 2 y 1 tras 3). En este trabajo se analizan los 34 que seguían sin tratamiento en el momento de los últimos datos conocidos. Se recogen los datos conocidos de cada paciente. Para su análisis se han dividido en dos grupos.

### 3.1 Grupo 1

Incluye los casos de los 23 pacientes en los que sólo se ha realizado un intento de retirada de FACE (Tabla 1).

**Tabla 1.** **Datos relativos a los 23 pacientes en los que sólo hubo un intento de retirada de fármaco anticrisis epilépticas (FACE), y que no toman FACE en último dato conocido**.

Caso.	Diagnóstico	Neuro-desarrollo	Edad primera crisis	Edad inicio FACE	FACEs tomados.	Número de crisis tomando FACE que finalmente se retiró.	Tiempo sin crisis hasta retirada	Tiempo sin FACE	Tiempo sin crisis
Sexo.					Tiempo tomando FACE hasta retirada en su caso				
Edad últimos datos									
1	Epilepsia con tipo de crisis desconocido y causa desconocida.	Normal	5m	5m tras 2 episodios de crisis repetidas en 10 días	VPT 3m	0	3m	2a3m	2a6m
Varón									
2a11m									
2	Epilepsia focal sintomática.	Normal	21m	25m	LEV no eficaz	Persisten crisis hasta extirpación quirúrgica con 3a1m.	5m	3a6m	3a11m
Mujer	Tumor neuroepitelial disembrioplásico temporal medial derecho.				CBZ 6m				
7a					VPT 5m				
3	Síndrome KBG por mutación *de novo* en gen *ANKRD11*.	DI	13m	13m tras crisis repetidas	LEV 22m	4	20m	3a	4a8m
Varón									
7a	Epilepsia sintomática con tipo de crisis desconocido.								
4	Síndrome de Usher.	DI + TEA	24m	24m tras crisis repetidas durante 2 días	LEV 3a3m	0	3a3m	22m	5a1m
Mujer	Hipoacusia.								
7a1m	Epilepsia con tipo de crisis desconocido y causa desconocida.								
5	Epilepsia con tipo de crisis desconocido y causa desconocida.	Normal	18m	19m tras repetidas crisis	LEV no eficaz ni tolerado	0 con VPT	VPT 22m	3a7m	*3m
Varón					VPT 22m				(Crisis tras 3a4m sin VPT)
7a3m									
6	Crisis febriles.	Normal	5a1m	6a3m tras segunda crisis	LEV 11m	0	11m	8m	19m
Mujer	Epilepsia con tipo de crisis desconocido y causa desconocida.								
7a10m									
7	Crisis febriles.	Normal	31m	4a1m tras crisis ocasionales	LEV 35m	Ocasionales durante 10m tomando LEV	24m	25m	3a1m
Mujer	Epilepsia focal causa no conocida.								
8a									
8	Crisis febriles.	Normal	3a10m	3a10m tras crisis diarias	LEV no eficaz no tolerado	0 con VPT	23m	23m	3a10m
Varón	Epilepsia con tipo de crisis desconocido y causa desconocida.				VPT 23m				
8a4m									
9	Epilepsia ausencias.	Normal	5a	6a1m tras ausencias diarias	VPT 23m	0	23m	10m	2a9m
Mujer									
8a10m									
10	Crisis febriles	Normal	Desconocido	19m	VPT 26m	Desconocido	Desconocido	5a4m	*25m.
Varón	Epilepsia con tipo de crisis desconocido y causa desconocida.								(Crisis tras 22m y tras 3a1m sin VPT.)
8a11m									
11	Variante *de novo* probablemente patogénica gen *PPP2R1A* (DI tipo 36, autosómica dominante)	DI	3a	3a tras primera crisis afebril, status 1 hora.	LEV 3a10m	3	24m	34m	4a10m
Mujer									
9a1m									
	Crisis febriles								
	Epilepsia focal causa no conocida								
12	Epilepsia ausencias	TDAH	4a5m	5a5m tras ausencias diarias	VPT 15m	0	15m	35m	4a10m
Varón									
9a7m									
13	Epilepsia con tipo de crisis desconocido y causa desconocida.	Normal	26m	26m tras crisis repetidas	LEV 4a8m	Ocasionales	24m	3a	4a11m
Mujer									
9a9m									
14	Epilepsia ausencias	Normal	6a8m	7a1m tras crisis diarias	VPT 24m	0	24m	15m	3a7m
Varón									
10a6m									
15	Deleción 17q21 31 síndrome de Koolen de Vries	DI	27m	33m tras tercer episodio de crisis afebriles repetidas.	VPT 27m	0	27m	5a8m	7a11m
Mujer	Crisis febriles								
10a8m	Epilepsia generalizada sintomática								
16	Epilepsia ausencias	Normal	6a10m	8a10m tras ausencias diarias	LTG 25m	0	24m	5m	29m
Varón									
11a4m									
17	Epilepsia focal causa no conocida	Normal	29m	VPT 35m	VPT no respuesta	3 desde OXC	35m	5a2m	7a1m
Varón 11a6m				OXC 3a7m	OXC 3a10m				
18	Epilepsia con tipo de crisis desconocido y causa desconocida.	Normal	9a11m	10a tras 2ª crisis	VPT 26m	Alguna crisis más floja	21m	21m	3a
Varón									
13a7m									
19	Epilepsia rolándica infancia	Normal	5a2m	10a9m.	CBZ persisten crisis	0 tras LEV	25m	9m	34m
Varón				Crisis hasta mensuales y hasta 4 misma noche, menores de 1 minuto, orofaciales o generalizadas. Nocturnas, salvo 1 despierto.	LEV 25m				
14a10m									
20	Crisis febriles.	DI	8a6m	8a7m tras 3ª crisis	VPT 17m	0	17m	6a4m	7a10m
Varón	Epilepsia con tipo de crisis desconocido y causa desconocida								
16a5m									
21	Epilepsia rolándica de la infancia	Normal	8a9m	8a11m	CLB no tolera	Ocasional	22m	5a6m sin OXC	2a8m
Varón 16a6m					ESL siguen crisis				
					OXC 27m				
22	Epilepsia generalizada	TDAH	11a11m	12a5m	VPT 17m	0	17m	2a8m	4a1m
Varón									
16a6m									
23	Epilepsia con tipo de crisis desconocido y causa desconocida, con todas las crisis durante el sueño.	Normal	14a	14a6m tras 3ª crisis	VPT 32m	1 tras 1a VPT	20m	4m	*1m
Varón 17a6m									(5ª crisis tras 3m sin VPT)

TDAH, trastorno por déficit de atención con/sin hiperactividad; TEA, trastorno del espectro autista; DI, discapacidad intelectual; VPT, ácido valproico; LEV, levetiracetam; CBZ, carbamacepina; LTG, lamotrigina; OXC, oxcarbacepina; CLB, clobazam; ESL, eslicarbacepina; *ANKRD11*, ankyrin repeat domain containing 11; *PPP2R1A*, protein phosphatase 2 scaffold subunit Aalpha. * En los casos 5, 10 y 23 no se reintrodujo FACE pese a crisis.

De ellos en 16 el neurodesarrollo es normal, 2 presentaban trastorno por déficit de atención con/sin hiperactividad (TDAH) aislado y 5 discapacidad intelectual (DI), en uno asociado a trastorno del espectro autista (TEA).

Se trata de 10 casos de epilepsia con tipo de crisis desconocido y causa desconocida, 4 epilepsias ausencias, 2 epilepsia rolándica de la infancia, 3 epilepsia focal de causa no conocida, 1 epilepsia focal sintomática, 1 epilepsia sintomática con tipo de crisis desconocido, 1 epilepsia generalizada sintomática y 1 otra epilepsia generalizada.

La edad media de los pacientes en el momento de los últimos datos conocidos es de 10,47 años, rango 2 años y 11 meses–17 años y 6 meses.

El primer fármaco instaurado fue ácido valproico (VPT) en 11 casos, levetiracetam (LEV) en 9 casos, lamotrigina (LTG) en 1, clobazam (CLB) en 1 y carbamacepina (CBZ) en 1.

La edad media de la primera crisis, excluido el caso 10 que no se conoce, es de 4,96 años, rango 5 meses–14 años.

La edad media en el momento de instauración del primer FACE es de 8,49 años, rango 5 meses–14 años y 6 meses.

El tiempo medio tomando el último FACE hasta retirada es de 26 meses, rango 3 meses–4 años y 8 meses.

8 niños presentaron crisis tomando el FACE que finalmente se retiró, incluido el caso 2 que presentó crisis hasta que se extirpó la tumoración. En el caso 10 se desconoce. Los otros 14 no tuvieron crisis tomando el FACE que finalmente se retiró.

El tiempo medio sin crisis hasta retirada del FACE, excluido el caso 10 que se desconoce, es de 21,13 meses, rango 3 meses–39 meses.

El tiempo medio sin FACE fue de 33,21 meses, rango 4 meses–6 años y 4 meses.

En 3 casos reaparecieron crisis, pero no se reintrodujo FACE: caso 23 que presentó 1 crisis tras 3 meses sin VPT, el caso 6 que presentó 1 crisis tras 3 años y 4 meses sin LEV y caso 9 que presentó 1 crisis tras 22 meses y otra tras 3 años y 1 mes sin VPT.

El tiempo medio sin crisis es de 44,78 meses, rango 1 mes–7 años y 11 meses. Si excluimos los 3 casos que presentaron crisis y sólo llevaban sin crisis 1 mes (caso 23), 3 meses (caso 6) y 25 meses (caso 9), el tiempo medio sin crisis es de 50 meses, rango 19 meses–7 años y 11 meses.

### 3.2 Grupo 2

Recoge los datos de los 11 pacientes en lo que se realizaron 2 o 3 intentos de retirada (Tabla 2).

**Tabla 2.** **Datos relativos a los 11 pacientes de la muestra a los que se ha retirado el tratamiento farmacológico en 2 ocasiones (9 casos, del 24 al 32) y 3 ocasiones (2 casos, 33 y 34), que no reciben tratamiento en el momento de los últimos datos conocidos**.

Caso.	Diagnóstico	Neuro-desarrollo	Edad primera crisis	Edad inicio FACE	FACEs tomados.	Intentos retirada FACE	Número de crisis tomando FACE que finalmente se retiró.	Tiempo sin crisis hasta retirada	Tiempo hasta nueva crisis desde retirada FACE	Tiempo sin FACE	Tiempo sin crisis
Sexo.					Tiempo tomando FACE hasta retirada en su caso						
Edad últimos datos											
24	Crisis febriles	Normal	22m	29m	VPT ineficaz y mal tolerado	2	0	LEV 3a	1m sin LEV	21m	*1m
Varón	Epilepsia generalizada				CLB mal tolerado			LTG 23m			2 crisis tras 20m sin LTG.
9a5m					LEV 3a						
					PER no tolerado						
					LTG 23m						
25	Síndrome de Panayiotopoulos	Normal	29m	4a11m	LEV 3a11m	2	0	21m 1ª vez	1m sin LEV	9m	2a11m
Mujer					LEV 26m			26m 2ª vez			
11a4m											
26	Crisis febriles	Normal	8a6m	8a11m	LEV	2	0	24m 1ª vez	1m sin LEV	20m	3a1m
Varón	Epilepsia generalizada				24m 1ª vez			27m 2ª vez			
13a11m					27m 2º vez						
**27	Epilepsia generalizada.	Normal	5a1m	5a2m	LEV 25m	2	0	25m LEV	2m sin LEV	4a8m sin VPT	6a8m
Mujer	Epilepsia ausencia.				*VPT 24m			24m VPT			
14a											
***28	Epilepsia ausencias	TDAH	7a6m	7a6m	VPT	2	0	13m	11m sin VPT dudosas ausencias	5a3m sin VPT	7a4m
Mujer					**13m 1ª vez			2m			
14a10m					**2m 2ª vez						
29	Epilepsia con tipo de crisis desconocido, de causa desconocida	Normal	4a4m	4a6m	VPT	2	0	25m 1ª vez	1 mes sin VPT	7a3m sin VPT	*6m
Mujer					4a5m 1ª vez			32m 2ª vez			1 crisis tras 6a9m sin VPT.
15a9m	Sincopes vagales, alguno convulsivo.				32m 2ª vez						
	TCA										
30	Crisis febriles	DI y TEA	3a	3a	VPT	2	Ocasionales	3a4m 1ª vez	7m sin VPT	3a sin VPT	8a7m
Varón	Epilepsia focal.				3a4m 1ª vez			6a 2ª vez			
18a3m	Mutación gen *KMT5B *MRD 51.				9a 2ª vez						
	Macrocefalia.										
31	Epilepsia mioclónica benigna del lactante.	TDAH	29m	29m	VPT	2	0	15m 1ª vez	5a7m sin VPT	6a5m sin VPT	9a4m
Mujer	Epilepsia generalizada genética.				15m 1ª vez			30m 2ª vez			
18a9m					30m 2ª vez						
****32	Epilepsia combinada generalizada y parcial, de causa desconocida.	Normal	13m	13m	VPT	2	0	30m VPT	3a sin VPT	12a10m sin LTG	14a7m
Mujer					CBZ			21m LMT			
28a8m	Epilepsia refractaria durante más de 9 años.				OXC						
					CLB						
					VGB						
					VPT 30m						
					LTG 21m						
33	Crisis febriles.	Normal	3a3m	3a11m	LEV	3	0	29m 1ª vez	1m sin LEV 1ª vez	3a5m sin LEV	5a5m
Mujer	Epilepsia rolándica de la infancia				29m 1ª vez			23m 2ª vez	1m sin LEV 2ª vez		
13a9m					23m 2ª vez			23m 3ª vez			
					23m 3ª vez						
34	Epilepsia con tipo de crisis desconocido, de causa desconocida	DI	5m	23m	VPT 8a1m	3	0	33m VPT	3a8m sin VPT	4m sin LEV	4a6m
Mujer					LEV			3a4m LEV	5m sin LEV		
20a6m	Microcefalia				3a4m 1ª vez			4a2m LEV			
					4a2m 2ª vez						

FACE, fármaco anticrisis epilépticas; TCA, trastorno de la conducta alimentaria; PER, perampanel; VGB, vigabatrina; *KMT5B*, lysine methyltransferase 5B; MRD 51, mental retardation, autosomal dominant 51. * En los casos 24 y 29 no se reintrodujo FACE pese a crisis. ** Tras recaída y reintroducción de LEV, aparecen ausencias que desaparecen al cambiar LEV por VPT. *** Las ausencias clínicas desaparecieron a los 2 días de tratamiento con VPT, pero manifestó claro empeoramiento de la atención con VPT y clara mejoría de la atención al retirarlo. Por electroencefalograma (EEG) con dudosas ausencias electroclínicas se reinició VPT, que se retiró otra vez a los 2 meses tras claro empeoramiento de la atención. Durante todo su seguimiento persistieron en el EEG descargas de paroxismos generalizados de grafoelementos punta-onda, de hasta 15 segundos de duración, sin traducción clínica clara. **** Datos actualizados al atender a su hijo por crisis febriles.

De ellos en 7 el neurodesarrollo es normal, 2 presentan TDAH y 2 DI, uno asociado a TEA.

Se trata de 5 casos de epilepsias generalizada, uno de ellos inicialmente crisis generalizadas y luego ausencias (caso 27), otro inicialmente como mioclónica benigna del lactante y luego crisis generalizadas (caso 31) y un caso sólo epilepsia ausencias, 2 epilepsia con tipo de crisis desconocido de causa desconocida, 1 epilepsia combinada generalizada y parcial de causa desconocida, 1 epilepsia rolándica de la infancia, 1 síndrome de Panayiotopoulos y 1 epilepsia asociada a mutación en gen lysine methyltransferase 5B (*KMT5B*).

La edad media de los pacientes en el momento de los últimos datos conocidos es de 16,28 años, rango 9 años y 5 meses–28 años y 6 meses. Si se excluye el caso 32, la edad media es de 15,05 años, rango 9 años y 5 meses–20 años y 6 meses.

En 7 pacientes el primer fármaco fue VPT y en 4 LEV.

La edad media de la primera crisis es de 3,62 años, rango 5 meses–8 años y 11 meses.

La edad media en el momento de instauración del primer FACE es de 4,15 años, rango 13 meses–8 años y 11 meses.

El tiempo medio tomando el último FACE hasta su retirada es de 2,9 años, rango 2 meses–9 años.

Sólo 1 niño (caso 30) de los 11, presentó crisis tomando el FACE que finalmente se retiró.

El tiempo medio sin crisis hasta la retirada del FACE es de 28,5 meses, rango 2 meses–6 años.

El tiempo medio hasta nueva crisis tras retirada del FACE es de 1,14 años, rango 1 mes–5 años y 7 meses.

El tiempo medio sin FACE fue de 4,3 años, rango 4 meses–12 años y 4 meses.

En 2 casos reaparecieron crisis, pero no se reintrodujo FACE: caso 24 que presentó 2 crisis tras 20 meses sin LTG y caso 29 que presentó una crisis tras 6 años y 9 meses sin VPT.

El tiempo medio sin crisis es de 5,7 años, rango 1 mes–14 años y 7 meses. Si excluimos los 2 casos que presentaron crisis y sólo llevaban sin crisis 1 mes (caso 24) y 6 meses (caso 29), el tiempo medio sin crisis es de 9,2 años, rango 35 meses–14 años y 7 meses.

## 4. Discusión

Cuando se retira el tratamiento farmacológico de las crisis epilépticas en niños, se debe tener asumido el riesgo de crisis por parte de médicos, padres y pacientes. La proporción de pacientes con recaídas durante o después de la retirada oscila entre el 12 y el 66% [1, 3]. En nuestra experiencia han vuelto al tratamiento farmacológico el 40% (23/57) de los casos en los que se había retirado, y el 38,9% (30/77) de los intentos de retirada.

Existe un consenso creciente de que la medicación debe suspenderse a partir de los 2 y 4 años sin crisis; nuestra tendencia está más cerca de los 2 años: el tiempo medio sin crisis hasta retirada del FACE es de 21 meses en el grupo 1 (rango 3–39 meses) y 28,5 meses en el grupo 2 (rango 2 meses–6 años).

La decisión de retirar el FACE debe ser individualizada, tras una adecuada información y conocimiento del riesgo de recaída.

Hay múltiples factores asociados de forma variable a un mayor riesgo de recurrencia de crisis: larga duración de la epilepsia antes de la remisión, intervalo corto sin crisis antes de la retirada del FACE, fallo en retirada previa, epilepsia de inicio en la adolescencia (11–17 años), edad avanzada de inicio de la epilepsia (en pacientes >25 años), historia familiar de epilepsia, historia de convulsiones febriles atípicas, historia de convulsiones neonatales, convulsiones parciales, convulsiones tónico-clónicas, crisis mioclónicas, múltiples tipos de crisis, afección neurológica subyacente, epilepsia sintomática remota, progresión tumor cerebral, epilepsia mioclónica juvenil, encefalopatía epiléptica, más de 10 convulsiones antes de la remisión, trastorno del neurodesarrollo, discapacidad intelectual, anomalía en la neuroimagen, anomalía epileptiforme en el EEG antes de la retirada (en niños) y politerapia [3, 4, 5, 6, 7, 8, 9, 10, 11].

También hay múltiples factores asociados de forma variable a la ausencia de crisis a largo plazo (10 años después del tratamiento antiepiléptico): corta duración de la epilepsia antes de la remisión, intervalo largo sin crisis antes de la retirada del FACE, mayor de 2 años, uno o dos fármacos antiepilépticos antes de la retirada, fácil control de las crisis con baja dosis de un FACE, epilepsia generalizada primaria como epilepsia ausencias (excepto la mioclónica juvenil), epilepsia “benigna” de la infancia (rolándica o síndrome de Panayiotopoulos), bajo número de crisis antes de la remisión, ausencia de antecedentes de crisis focales, exploración neurológica normal, ausencia de actividad epileptiforme en el electroencefalograma (EEG) antes de la retirada [3, 4, 5, 6, 7, 8, 9, 10, 11].

La indicación de inicio o retirada de un FACE debe considerar en primer lugar que el tratamiento farmacológico de la epilepsia no es curativo, pretende evitar las crisis o bien disminuir su frecuencia, intensidad y duración [12], que el pronóstico de la epilepsia, en general, no cambia por la instauración precoz de tratamiento con FACE [13], no está demostrado que la retirada de los FACE afecte negativamente a los resultados de las crisis a largo plazo en pacientes que dejaron de tener crisis bajo el tratamiento con FACE [14, 15, 16], y parece que el resultado de las crisis a largo plazo no se ve afectado por la interrupción del fármaco [6, 7, 8].

No hay evidencias respecto a la evitación del riesgo de muerte súbita inesperada por epilepsia con FACE, y en la actualidad su empleo para prevenirlo en niños con crisis ocasionales no está justificado [17].

La continuación del tratamiento tampoco garantiza la ausencia de crisis, como consecuencia de la progresión de la epilepsia [5, 15]. En nuestra experiencia habían presentado crisis tomando el FACE que finalmente se retiró el 27,27% (9 de 33) de los pacientes.

Este trabajo recoge la experiencia con niños epilépticos durante muchos años, en los que hemos ido modificando nuestra forma de actuar adaptándonos a una crítica evaluación de los conocimientos y experiencias.

La decisión de mantener o retirar un FACE se realiza de forma individualizada, según el riesgo de recaída de crisis y potencial repercusión sobre la vida diaria, junto a la percepción de eficacia y tolerancia del FACE. En la mayoría de los casos no se ha podido establecer retrospectivamente los datos de eficacia y tolerancia, por lo que no se han podido analizar de forma fehaciente, siendo conceptos habitualmente relativos que dependen del nivel de vigilancia de la evolución de cada paciente.

En nuestra serie se dejaron sin FACE casos con riesgo alto de seguir presentando crisis como 7 casos de discapacidad intelectual (2 de ellos con TEA asociado), epilepsia refractaria en 1 caso (caso 32), fallo en retirada previa en 11 niños y epilepsia de inicio en la adolescencia en dos casos: a los 11 años y 11 meses en el caso 22 y a los 14 años en el caso 23.

Creemos que, pese al mayor riesgo de crisis, debe valorarse un intento de retirada en adolescentes antes de pasar a control a neurología de adultos, ya que en adultos puede ser más difícil, por la existencia de factores que no se dan en niños, como permiso de conducir o trabajo.

Siempre hemos sido muy estrictos en mantener un FACE sólo si es eficaz, y habitualmente nuestros pacientes han podido estar tomando simultáneamente máximo 2 FACEs o en algún caso 3 (cortos períodos).

Cada vez hay más evidencias de que muchas epilepsias asocian comorbilidades, especialmente cognitivas y conductuales, que precisan un manejo adecuado [18]. En nuestra serie, de los 34 casos, 4 presentaban TDAH aislado y 7 DI, asociado a TEA en 2 casos.

Existe una mayor mentalización sobre la potencial repercusión negativa de los FACE sobre el neurodesarrollo (más a más pequeño sea el niño) y aspectos de función ejecutiva como atención, comportamiento o rendimiento escolar. Cualquier FACE en muchos pacientes, y con frecuencia los más prescritos en pediatría, LEV y VPT, pueden causar o empeorar síntomas de disfunción ejecutiva cerebral, como déficit de atención, hiperactividad e impulsividad, muy prevalentes en la población pediátrica [18, 19, 20, 21, 22, 23].

Nuestra preocupación por la repercusión de los FACE sobre el neurodesarrollo y las funciones ejecutivas se ha ido incrementando en la última década [22, 23]. En esta serie el FACE inicial fue VPT en 18 casos y LEV en 13 casos, lo que refleja nuestra pauta de actuación en el pasado. En los últimos años utilizamos habitualmente LTG de primera elección en cualquier tipo de epilepsia pediátrica si no existe urgencia en el control de las crisis, por presentar en general, menor repercusión sobre función ejecutiva, comportamiento y neurodesarrollo [22].

El estudio presenta como limitación principal su carácter retrospectivo, con una serie pequeña y heterogénea. Destacamos como positivo el poder recoger la evolución de nuestra acción clínica, siempre en permanente replanteamiento crítico, buscando el beneficio de los niños. Esto evita el sesgo de los estudios prospectivos, que difícilmente podría disponer de una serie homogénea y amplia de pacientes pediátricos con epilepsia a los que se ha retirado tratamiento.

Defendemos la importancia de las revistas médicas como herramienta de comunicación y discusión de las acciones clínicas en la vida real.

## 5. Conclusión

La indicación y el mantenimiento del tratamiento con FACE en niños debe priorizar el bien del paciente, por encima de miedos o zonas de confort, y deben estar basados en tres premisas: que esté indicado (lo cual en algunos casos puede ser discutible), que sea tolerado (especialmente neurodesarrollo y funciones ejecutivas) y que sea eficaz.

Si un FACE no es tolerado debe retirarse y buscar otra opción, y si no es eficaz debe cambiarse.

La calidad asistencial exige un esfuerzo continuado por adecuar las intervenciones a la mejor respuesta y tolerancia en un proceso de mejora que no tiene punto final. En el caso de los FACE en niños, tiene especial relevancia la vigilancia de su repercusión sobre el neurodesarrollo, las funciones cognitivas, la atención y el comportamiento.

## Data Availability

Los datos analizados durante el estudio actual no están disponibles públicamente debido a confidencialidad.

## Contribuciones de los Autores

JLP diseñó el estudio de investigación. JLP, CGS y PMC realizaron la investigación. JLP, MVFJ, FMC y RFM analizaron los datos. JLP, MVFJ, FMC y RFM redactaron el manuscrito. Todos los autores contribuyeron a los cambios editoriales en el manuscrito. Todos los autores leyeron y aprobaron el manuscrito final. Todos los autores han participado lo suficiente en el trabajo y han acordado ser responsables de todos los aspectos del mismo.

## Aprobación Ética y Consentimiento Informado

El protocolo de investigación fue aprobado por el Comité de Ética de Aragon Clinical Research Ethics Committee (CEICA) (Número de Aprobación Ética: EOM24/032). Para garantizar la anonimización de los pacientes, no se incluye ningún dato de identificación y se asigna un código numérico a cada paciente, diferente del número de historia clínica, que solo conocen los investigadores. Basándose en esta anonimización, el Comité de Ética eximió el consentimiento informado. El estudio se llevó a cabo de acuerdo con las directrices de la Declaración de Helsinki.

## Agradecimientos

No aplicable.

## Financiación

Esta investigación no recibió financiación externa.
